# The role of parietal cortex in overimitation: a study with fNIRS

**DOI:** 10.1080/17470919.2017.1285812

**Published:** 2017-02-07

**Authors:** Dominic Oliver, Ilias Tachtsidis, Antonia F de C Hamilton

**Affiliations:** ^a^ Institute of Cognitive Neuroscience, University College London, London, UK; ^b^ Medical Physics and Biomedical Engineering, University College London, London, UK

**Keywords:** fNIRS, parietal cortex, imitation, action observation

## Abstract

Previous studies have shown right parietal activation in response to observing irrational actions. Behavioral studies show that people sometimes imitate irrational actions, a phenomenon called overimitation. However, limitations on movement in functional magnetic resonance imaging (fMRI) mean that the neural basis of overimitation has not been studied. To address this, our study employed a less restrictive neuroimaging technique, functional near-infrared spectroscopy (fNIRS). Measurements were taken while participants observed either rational or irrational movements before performing movements on a computerized puzzle task. Observing irrational actions produced greater activation in right anterior inferior parietal lobule (aIPL), replicating results from the fMRI literature. This is a proof of principle that fNIRS can be used as an alternative to fMRI in social cognition experiments, and that parietal cortex has a core role in responding to irrational actions.

## Introduction

Imitation is an important human behavior, which allows us to learn new skills and to connect with others (Uzgiris, 1981). Recently, there has been increased interest in understanding the mechanisms and motivations that determine imitative fidelity (Over & Carpenter, 2012). There are many circumstances in which children and adults imitate other people’s actions with very high fidelity, even when this compromises the efficiency of their own actions (Horner & Whiten, 2005; Lyons, Young, & Keil, 2007), a phenomenon termed “overimitation.” This paper examines the neural mechanisms supporting imitation and overimitation in the adult parietal cortex, using a novel functional near-infrared spectroscopy (fNIRS) method.

### Overimitation behavior

Overimitation has typically been studied in young children, who are shown a sequence of actions including both rational actions (that help achieve the goal state) and irrational actions that do not contribute to the goal. For example, an adult demonstrates a sequence of actions to take a toy out of a complex puzzle box, including some irrational or irrelevant actions in the sequence. The child is then instructed to remove the toy from the box as fast as possible. Despite the explicit time constraints, children still imitated both the rational and irrational actions (e.g., the demonstrator stroking the box with a feather), even though the irrational actions were clearly unrelated to the goal (Horner & Whiten, 2005; Lyons et al., 2007).

Overimitation does not represent simply a failure of young children to understand the relevance of actions, as overimitation has been shown to increase with age (McGuigan, Makinson, & Whiten, 2011; McGuigan, Whiten, Flynn, & Horner, 2007) and is unrelated to familiarity of object or goal (Marsh, Ropar, & Hamilton, 2014a). Recent explanations of overimitation have focused on the social nature of this behavior, as a sign of social affiliation or conformity to a social norm (Kenward, Karlsson, & Persson, 2010; Marsh, Pearson, Ropar, & Hamilton, 2013). Social cues, such as demonstrator status, have been shown to modulate overimitation. For example, it has been shown that children will only reproduce irrational actions when being observed by the demonstrator (Lyons et al., 2007; Nielsen & Blank, 2011). This suggests that overimitation is an important social behavior, and motivates us to explore its neural mechanisms.

### Neural mechanisms of imitation and rationality detection

Two major brain networks have been highlighted in previous neuroimaging studies of action rationality and imitation studies. First, the human mirror neuron system (MNS) in the inferior parietal and inferior frontal lobes is strongly linked to the observation and imitation of actions (Caspers, Zilles, Laird, & Eickhoff, 2010; Rizzolatti & Sinigaglia, 2010). Second, the medial prefrontal cortex (mPFC) and temporoparietal junction (TPJ) (mentalizing network) have a role in comprehending complex or irrational actions (Brass, Schmitt, Spengler, & Gergely, 2007; Spunt & Lieberman, 2012).

Observed actions are somatotopically arranged in both premotor cortex and parietal lobule, suggesting these areas are likely key in processing, observing a demonstrator’s actions (Caspers et al., 2010; Molenberghs, Cunnington, & Mattingley, 2009). The rostral inferior parietal cortex is thought to form flexible representations of observed sequences that can be applied to various different effectors (Grafton, Hazeltine, & Ivry, 1998). Imitating novel actions compared with familiar actions is linked to increased activity in bilateral superior parietal cortex and right parieto–occipito junction (Rumiati et al., 2005). In combination, these studies suggest that parietal cortex may have a role in high fidelity imitation.

While none of these studies have explicitly considered how imitation might be modulated by action rationality, several papers do show differences in brain activation for observation of rational and irrational actions. First, Brass et al. (2007) showed participants videos of people performing unusual actions (e.g., turning on a light switch with the knee) in a context where the action was rational (because the actor’s hands were occupied carrying folders) or irrational (the actor’s hands were free). Observing irrational actions led to increased brain activation in mPFC and posterior superior temporal sulcus (STS) compared with the rational condition. A follow-up study contrasting intentional or accidental irrational actions found greater mPFC engagement for intentional irrational actions (Desmet & Brass, 2015). These areas are linked to higher-order visual processing and mentalizing, rather than mirror systems, suggesting that understanding irrational actions may require more than a direct mapping between sensory and motor systems.

Action rationality can also be defined by the curvature of a hand trajectory. Typical hand actions are efficient and proceed in a near-straight line from the start to end point (Abend, Bizzi, & Morasso, 1982), and infants from 9 months of age can discriminate straight actions from irrational curved actions which do not avoid an obstacle (Csibra, Gergely, Bíró, Koós, & Brockbank, 1999). Observation of rationality defined by straight and curved trajectories has been tested in three studies. Jastorff, Clavagnier, Gergely, and Orban (2011) found that activity in middle temporal gyrus (MTG) correlated significantly with action rationality in observation of actions of varying curvature. These results contrast with two studies from Marsh et al., despite a similar definition of rationality. In both, observation of irrational actions led to increased activation of right parietal cortex, particularly intraparietal sulcus, and to reduced activation of mPFC (Marsh & Hamilton, 2011). In the second study, right inferior parietal lobule (IPL) and TPJ were also engaged when observing irrational actions (Marsh, Mullett, Ropar, & Hamilton, 2014c).

Together, these studies suggest that observation of irrational actions engages regions of the MNS, in particular right IPL, but also higher-order visual regions (STS and MTG) and mentalizing regions (TPJ and mPFC). However, none of these studies required participants to imitate the irrational actions, and thus the neural mechanisms of overimitation remain unclear. It is important to note that it is very hard to produce large or complex actions in functional magnetic resonance imaging (fMRI) without seriously harming the quality of data. One study attempted to circumvent this by having participants watch videos of objects being assembled while in an fMRI scanner and had to imitate the sequence afterwards outside the scanner. MNS areas were more active when participants intended to learn the action sequence compared with the perceptual control condition. Right parietal activity during observation predicted later imitation fidelity, suggesting a key role in high fidelity imitation (Frey & Gerry, 2006). However, performing natural hand actions in fMRI with full visual feedback remains very hard to implement. In order to study imitation mechanisms, we needed to record brain activation while participants can perform actions freely. fNIRS provided a means to achieve this.

### The fNIRS methodology

fNIRS is a low-cost, safe, noninvasive neuroimaging technique that can measure blood oxygenation and hemodynamics in the cortical surface just below the skull. fNIRS measures the changes in concentrations of oxygenated and deoxygenated hemoglobin (oxy-Hb and deoxy-Hb, respectively), which relate to neural activity in the same way as the BOLD signal used in fMRI. When an fNIRS measurement is taken over an area where cerebral blood flow increases in response to increased brain activity, a marked increase in oxy-Hb should be seen alongside a decrease in deoxy-Hb (Tachtsidis & Scholkmann, 2016). fNIRS has one major advantage over both fMRI and EEG – it is very robust to movement, allowing participants to move their heads and bodies naturally as they perform everyday cognitive tasks. Recent data show fNIRS can be used during walking (Pinti et al., 2015) and dancing (Noah et al., 2015), yielding comparable results to fMRI (Noah et al., 2015). For this reason, fNIRS is well suited to the study of imitation and social behavior. For a detailed review of the method, see Scholkmann et al. (2014).

### Current study and hypotheses

We needed to employ a behavioral task that firstly shows overimitation in adults and is amenable to neuroimaging (e.g., multiple trials with a controllable timing pattern). Typical adults have been found to rate curved actions as irrational compared with straight actions when there are no obstacles (Marsh, Pearson, Ropar, & Hamilton, 2014b). When asked to copy a sequence of pointing movements to different dot locations, typical adults overimitated, copying the curvature of the demonstrated action, despite high curvature actions being less rational and no explicit instruction to copy curvature (Wild, Poliakoff, Jerrison, & Gowen, 2009). Therefore, we elected to move away from puzzle boxes and used trajectory curvature as way to explore imitation and overimitation in typical adults.

In the present study, we asked participants to perform a simple picture-building task, in a context where they could imitate the curvature of a demonstrator’s action if they chose to. This allowed investigation of overimitation behavior as well as recording neural data from parietal cortex. We predicted that the right TPJ and IPL would be engaged when observing irrational actions compared with observing rational actions, as seen in Marsh et al. (2014c), and these areas will be more strongly activated when imitating irrational actions.

## Methodology

### Participants

Healthy participants aged 18–39 years old were recruited from the Institute of Cognitive Neuroscience (ICN) Subject Database. Thirty participants attended recording sessions but due to either thick hair or a poorly fitting fNIRS cap, data could only be recorded from 18. Further exclusions due to spatial deviations and anomalous data left a final sample of 14 participants (10 female, mean age 25.07, SD 6.39).

All participants were right-handed and had normal-to-corrected vision (self-report). Participants had no known neurological or psychiatric conditions. Full written consent was given by participants to take part in the study. The study was granted full ethics approval by the ICN Research Department’s Ethics Chair (Project ID No: **Z6364106/2014/02/21**). Participation lasted approximately an hour and a half, for which participants were paid £11.25 (£7.50 per hour).

### Equipment and preparation

Participants were seated 860 mm from a projector screen (2032 × 1143 mm) on a wooden stool with a wooden table. Participants performed the task using a wireless mouse and keyboard. An Artinis Medical Systems Oxymon Mk III fNIRS system and OxySoft software were used to collect data. A National Instruments USB-6001 DAQmx was used to synchronize the time signatures of task events with the fNIRS data.

Electroencephalogram (EEG) caps in different sizes (from EasyCap) were customized to hold the optode arrays over the parietal cortex. The fNIRS system optodes were arranged in two 2 by 2 arrays. Each array contained two illuminating optodes and two detecting optodes, spaced 3.5 cm apart. This provided four channels per array, totaling eight channels across the head. Oxy-Hb and deoxy-Hb were measured using fNIRS with two continuous wavelengths of light at 760 and 850 nm. The sampling rate was set at 10 Hz.

#### Spatial registration of fNIRS channels

**Figure 1.**
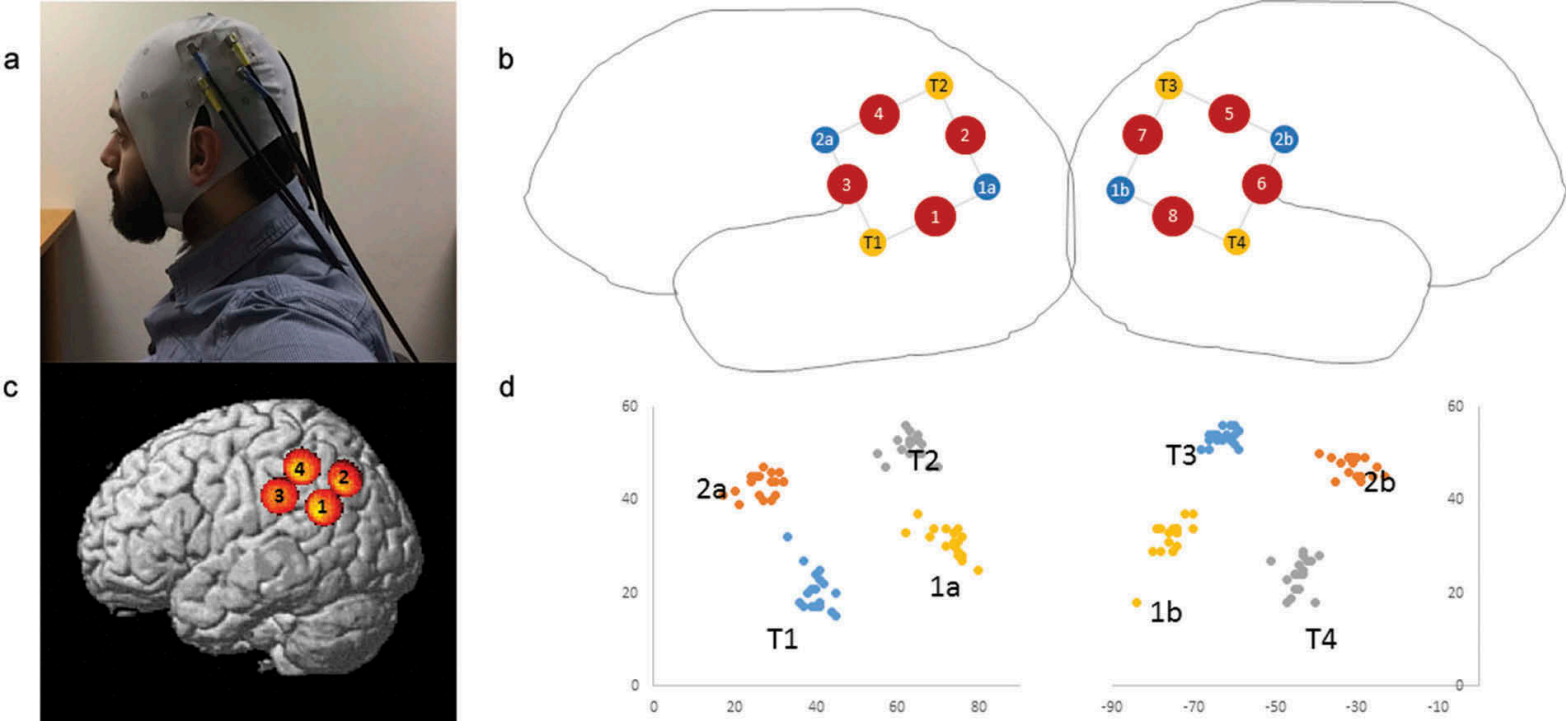
a) Photo of custom fNIRS cap set up b) Schematic showing layout of light-emitting optodes (yellow circles), light-detecting optodes (blue circles), and channels (red circles) c) Average left hemisphere channel locations across all participants see Table 1 d) Scatter plot of y and z coordinates of optodes for each individual participant to show variability due to cap placement.

For each participant, head circumference was measured to determine the correct cap size and vertex was marked on the scalp with a washable marker. The cap was placed with Cz on vertex so the two optode arrays were positioned over parietal cortex (see Figure 1(a,b)). Before data recording began, we digitized the location of each optode using a Polhemus Liberty magnetic motion tracker. One Polhemus marker was fixed to the forehead with a Velcro strap, while a second marker was used to point in turn to each of the five canonical head locations (nasion, inion, vertex, left auricular, right auricular) and to each of the eight optodes. The position and orientation of both markers was saved for each location using a custom MATLAB® script.

After recording, a custom MATLAB® script converted the Polhemus position/orientation data for each marker to an Affine matrix representing the transformation from the origin to the marker. Standard affine transforms were then used to represent the position of each recorded position (five canonical head locations + eight optodes) in relation to the marker fixed on the forehead. This provides a robust correction for any head movement during the recording procedure, and ensures high quality localization data. These standardized locations were used as inputs into the Near Infrared Spectroscopy-Statistical Parametric Mapping (NIRS-SPM) interface to normalize them to a canonical brain (Ye, Tak, Jang, Jung, & Jang, 2009) and obtain the Montreal Neurological Institute (MNI) coordinates of each optode (Table 1).

**Table 1.** Significant results from *t*-tests in the trial-level analysis.

Channel number	MNI	Neural localization	S > B	B > S	C > B	B > C	S > C
1	−61–57 26	Left TPJ					DX: *t* = 2.395, *p* = 0.032
2	−52–68 41	Left angular gyrus					
3	−67–33 32	Left anterior IPL					
4	−57–45 47	Left posterior IPL			TX: *t* = 2.28, *p* = 0.04	DX: *t* = −2.636, *p* = 0.021	
5	58–47 50	Right posterior IPL	TX: *t* = 2.503, *p* = 0.026		OX: *t* = 3.068, *p* = 0.009, TX: *t* = 3.646, *p* = 0.003		
6	67–37 36	Right anterior IPL					
7	50–69 43	Right angular gyrus	TX: *t* = 2.551, *p* = 0.024		OX: *t* = −3.044, *p* = 0.009; TX: *t* = 3.341, *p* = 0.005		
8	60–60 28	Right TPJ	TX: *t* = 2.522, *p* = 0.026	DX: *t* = −2.563, *p* = 0.024	OX: *t* = 2.23, *p* = 0.044; TX: *t* = 2.53, *p* = 0.025		

OX: oxy-Hb; DX: deoxy-Hb; TX: total-Hb.

**Figure 2.**
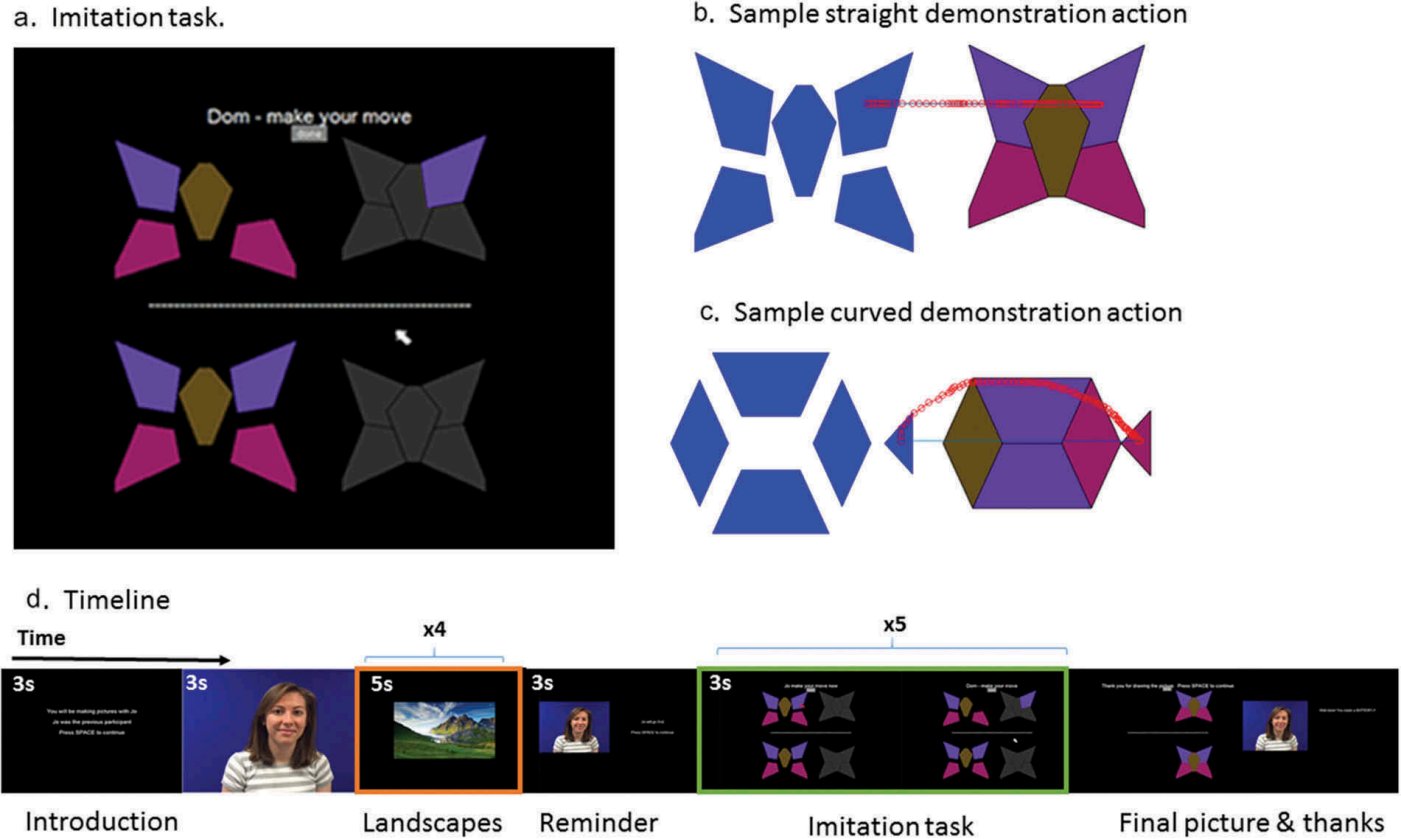
a) Screen during the imitation task. The five puzzle pieces on the top half belong to the demonstrator, and the five on the lower half to the participant. b) and c) illustrate sample straight and curved actions used by the demonstrator to move a piece from left to right to build the picture. d) Illustrates the study timeline. First, participants read instructions and saw a video of the demonstrator who was described as a previous participant. They completed trials of the imitation task with five turns in each trial (green box). Reminders of the demonstrator were presented before each imitation trial and thanks after each trial to enhance the social feeling. Between imitation trials, participants viewed landscape pictures (orange box).

Plots of the MNI coordinates obtained for each participant were created (Figure 1(d)) to assess the variance of optode placement across participants. For three participants, the recorded optode locations deviated from the group, suggesting that the recordings for these individuals would not pick up the same brain regions as the rest of the group. Thus, these three were excluded. For the remaining 14 participants, fNIRS channel locations were calculated as the midpoint between the group-average optode locations. Channel locations were then visualized in SPM (Figure 2(c)) to allow us to label the brain regions that were the focus of each fNIRS channel. The final MNI coordinates of the channels and their anatomical names are given in Table 1.

### Task procedure

Participants completed a cognitive task implemented in MATLAB® and Cogent Graphics, which was designed to allow possible imitation of irrational curved actions. Participants were told they were to complete a “picture-building” task with another person. At the start of a trial, participants could see an array of shapes on the screen (Figure 2(a)). In the top left quadrant of the screen were five colored puzzle pieces for the demonstrator to move; in the top right quadrant were five gray outlines indicating where the puzzle pieces should be placed. In the lower left quadrant of the screen were five colored puzzle pieces for the participant to move; in the lower right quadrant were five gray outlines indicating where the pieces should go. Each puzzle piece could be picked up by clicking with the mouse, moved to another location, and put down by clicking.

The sequence of events in an observation/imitation trial was as follows. Participants would see an instruction screen “you will make a fish” followed by a screen showing puzzle pieces as described above. They would see a red colored mouse cursor pick up one piece from the demonstrator’s left quadrant and move it over to the appropriate location in the demonstrator’s right quadrant. When the demonstrator’s turn was finished, the mouse cursor turned white and the participant could move a piece from her left quadrant to her right quadrant. Participants were instructed to click a “done” button near the top of the screen when their turn was over. The demonstrator then moved another piece for another turn. A total of five demonstrator turns and five participant turns were required to complete the picture. Then a positive feedback screen was shown before the next trial. The word “imitation” or “copy” was never used, and the task was presented to participants as a goal-directed “picture making” task rather than an imitation task.

The mouse actions of the demonstrator were prerecorded movements of a single person performing the same task under two different instructions. For “straight” actions, the demonstrator moved the mouse from left to right in a horizontal line across the screen. For “curved” actions, the demonstrator moved the mouse from left to right with a distinct upward curvature (see Figure 2). The primary imitation measure on the current task is whether participants show the same curvature pattern as the demonstrator. To assess this, the complete mouse trajectory and clicks on every turn were recorded.

Over a single experimental run, participants completed nine pictures in a randomized order. Curvature was manipulated between pictures (e.g., all five demonstration movements for the fish picture would be curved, while all five for the cat picture were straight) and counterbalanced between participants. Each trial of the puzzle task lasted approximately 60 s. To increase social engagement, participants were introduced via a prerecorded video to the demonstrator, Jo, who they were told was the previous participant (Figure 2). After each puzzle trial, participants would be congratulated on completing the previous trial “with Jo” and saw a picture of Jo. In between puzzle trials, participants spent 20 s viewing landscape pictures (four pictures shown for 5 s each) and were instructed to relax. This condition provides a baseline where we expect no activation in parietal cortex related to action production, action observation, or imitation. All participants completed two experimental runs, each lasting around 15 min.

To align behavioral and fNIRS timelines, analog signals were sent from the task computer using a National Instruments USB-6001 DAQmx and were recorded on the analog input channel of the Artinis fNIRS system in sync with the fNIRS signals. Different signal magnitudes (0 V, 1 V, 2 V, etc.) were used to mark the start of a straight trial, curved trial, or baseline trial, and to signal the start and end of turns within each trial.

### Data analysis

#### Behavioral data analysis

Mouse data were processed through a custom MATLAB® script. The complete mouse trajectory for each turn the participant completed was separated into the time periods when the participant moved a puzzle piece. Manual data inspection was used to exclude turns where the participant failed to drop the piece at the right time or with an incomplete or abnormal movement. For the valid movements, movement distance was calculated as the difference between the x–y coordinates of the first mouse click (lifting the piece) and the x–y coordinates of the second mouse click (placing the piece). Movement height was calculated as the maximum orthogonal distance of the mouse trajectory from a line joining the first and second mouse clicks. Curvature was defined as height/distance (Figure 2(c)). The curvature of the demonstrator’s movements ranged from 0.02 to 0.3. Any participant curvature values greater than double the maximum demonstrated movement were excluded from the analysis. Mean curvature values for each condition were calculated across participants. Curvature could be compared across conditions and to the curvature of the demonstrator’s movements in these conditions.

#### fNIRS data analysis

Immediately after data recording, the raw fNIRS data were converted into concentrations of oxy-Hb, deoxy-Hb, and total Hb using the modified Beer–Lambert Law as implemented by the OxySoft software package. The Beer–Lambert Law was intended to be applied to a clear, nonscattering medium. When being applied to biological tissue, a differential pathlength factor (DPF) must be incorporated to account for increased optical pathlength caused by scattering. In this study, a fixed DPF of four was used. These data were then exported for analysis in MATLAB® using a combination of custom MATLAB® scripts and the NIRS-SPM package (Ye et al., 2009). When the data were imported into MATLAB®, it was visually inspected for movement artifacts caused by cap slippage and data sets affected were excluded from analysis (*n *= 1).

A fourth-order low-pass Butterworth filter with a cutoff frequency of 0.1 Hz was applied to remove high frequency fluctuations. Total Hb was calculated as the sum of oxy-Hb and deoxy-Hb. To simplify scripting, data for all three Hb parameters (oxy-Hb, deoxy-Hb, and total Hb) and all eight channels were concatenated into a single 24 × n data matrix where n is the number of time points recorded. For typical neural activation, fNIRS records an increase in oxy-Hb and a concurrent decrease in deoxy-Hb (Tachtsidis & Scholkmann, 2016). Total Hb similarly increases during activation.

For each participant, two design matrices were built to examine the role of parietal cortex in imitation of straight and curved actions. The first modeled data only on a trial level, considering both observation of the demonstrator’s action and performance by the participant together. The second examined the data on a turn-by-turn basis, modeling each turn from the demonstrator and the participant separately. Details of each are given below.

#### Trial level analysis

This design matrix had three regressors for each of the three conditions: straight trials, curved trials, and baseline. A box car for each regressor was convolved with the canonical haemodynamic response function, the temporal derivative and the dispersion derivative. The design matrix was then estimated to obtain beta parameters for each run. Because most participants completed two runs, the beta parameters for each person were averaged across the two runs. At the second level of analysis, beta parameters for each Hb signal and each channel were compared against each other using paired *t*-tests.

#### Turn level analysis

This design matrix had five regressors. The first four made up a 2 × 2 factorial design with factors of role (observing the demonstrator action/performing an action) and movement (straight/curved) for each turn in the experiment. The fifth regressor was the baseline trials as before. First-level analysis was as described in trial-level analysis. At the second level of analysis, beta parameters for each Hb signal and each channel were compared against each other using ANOVA and paired *t*-tests.

#### Correction for multiple comparisons

In the present study, we contrasted five tests (main effects and interaction in a 2 × 2 ANOVA, plus two comparisons to baseline) in eight channels and three signals (oxy-/deoxy-/total-Hb), which presents a large number of comparisons. Applying a Bonferroni correction across such data would be very harsh, but without any correction for multiple comparisons, we run a high risk of false positives. Instead, we take advantage of our knowledge of sources of variability in fNIRS data to develop a more appropriate correction.

Neural activity leads to two specific changes in fNIRS data – an increase in oxy-Hb and a parallel decrease in deoxy-Hb. However, the fNIRS signal may also pick up many other physiological changes including changes in blood flow due to heartbeats, breathing, and blood pressure. Such physiological changes are positively correlated between the oxy-Hb and deoxy-Hb signals (i.e., an increase in both or decrease in both) (Tachtsidis & Scholkmann, 2016). Noise in the signal can also be introduced by the interface between the optodes and the scalp, and the fiber optics and detectors themselves. Such noise is uncorrelated or positively correlated between oxy-Hb and deoxy-Hb signals. Knowing these error sources, we can restrict our search for neural activation to only those channels, which show both an increase in oxy-Hb and a decrease in deoxy-Hb – the signature of neural activation.

To implement this, we set a per-channel threshold of θ and require that a channel must show a positive effect with *p* < θ in the oxy-Hb signal AND a negative effect with *p* < θ in the deoxy-Hb signal in order to count as significant. This is equivalent to a true fMRI conjunction analysis (Nichols, Brett, Andersson, Wager, & Poline, 2005). With this criterion, the probability that a significant effect arises by chance in a single channel is θ^2^, so over the whole array, we require θ^2^ < 0.05/*n* which includes a Bonferroni correction for *n* channels. Solving to find θ gives θ = sqrt(0.05/*n*), and setting *n* = 8 × 5 for the number of comparisons (eight optodes × five contrasts), shows that our per-channel threshold should be 0.0791. A Monte Carlo simulation confirms this value. Thus, we used a *p* < 0.0791 per-channel threshold with the additional requirement that we find a positive effect in the oxy-Hb signal and a negative effect in the deoxy-Hb signal in the same channel. This provides a robust correction for multiple comparisons that takes into account the characteristics of fNIRS data. Using this threshold instead of the more common *p* < 0.05 threshold did not garner more results replicated across oxy- and deoxy-Hb, highlighting how robust these data are to various thresholds.

## Results

### Behavioral mouse data

**Figure 3.**
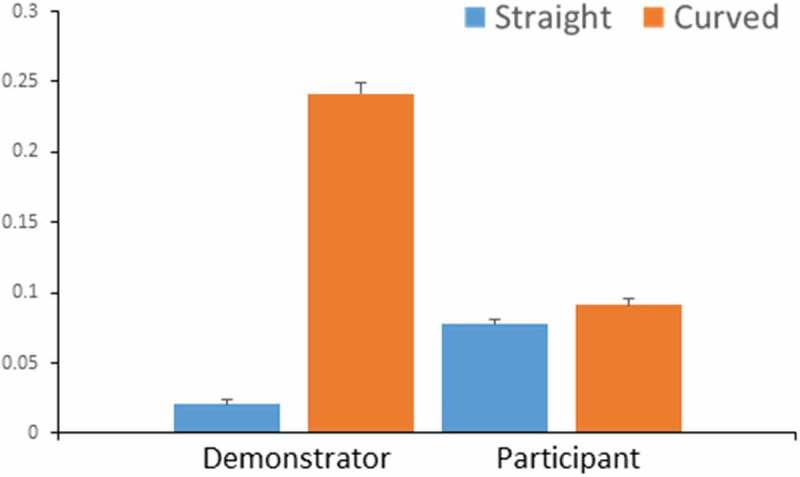
Graph comparing curvature of participants’ movements (blue) compared to the demonstrator’s (orange) in rational straight condition and in irrational curved condition. Curvature of participants’ movements was significantly different between conditions (*t*(17) = −2.836, *p* = 0.005).

Mouse data were analyzed to assess participants’ overimitation in the irrational curved condition. We expected that participants would produce straight movements after observing rational actions and produce curved movements after watching irrational actions. Curvature was calculated as the divergence from the most efficient straight movement from the piece to the template. There were significant differences between the curvature of participants’ movements in the straight condition compared with the curved condition (*t* (17) = −3.270, *p* = 0.005) (Figure 3), which confirms that participants imitated the actions they saw. However, this imitation effect was weak, and the curvature of participants’ movements in the straight condition was significantly higher than the demonstrated movements (*t* (36) = −11.153, *p* < 0.001) and significantly lower in the curved condition (*t* (36) = −16.946, *p* < 0.001) (Figure 3). There was no significant effect of scene order on curvature in either rational straight (*t* (17) = 1.874, *p* = 0.079) or irrational curved conditions (*t* (17) = −0.633, *p* = 0.536).

### Trial-level analysis

No results met our corrected thresholds with effects in both oxy- and deoxy-Hb channels for the trial-level analysis. Marginal effects are reported in Table 1.

### Turn-level analysis

Turn-level data were first analyzed with a repeated measures ANOVA to test for main effects of rationality, main effects of observation/performance and rationality by observation/performance interactions. Second, observation of action was contrasted to the landscape baseline, and performance of action was contrasted to the landscape baseline.

**Figure 4.**
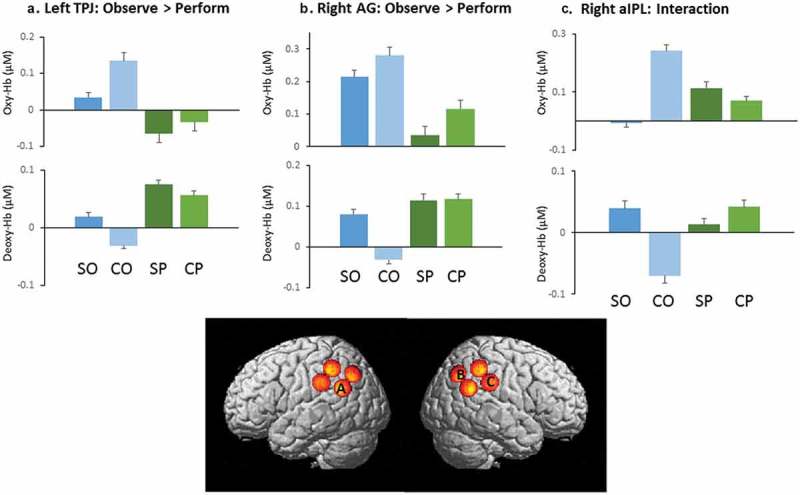
Brain areas with significant effects seen across oxy- and deoxy-Hb. Bars refer to levels in straight observe, curved observe, straight perform, curved perform, and baseline conditions, respectively. Note that typical effects show an increase in oxy-Hb and a decrease in deoxy-Hb.

Left TPJ and right angular gyrus displayed an increase in oxy-Hb with a concurrent decrease in deoxy-Hb (Figure 4(a,b)) when participants observed actions compared with when they performed actions. This indicates greater neural activity for the observation of actions compared with performance. Right anterior IPL (aIPL) showed an interaction between rationality and performance, such that there was an oxy-Hb increase/deoxy-Hb decrease when participants observe irrational actions but not when they perform irrational actions. This indicates selective neural activation for the observation of irrational actions only (Figure 4(c)). Marginal effects are reported in Table 2.

**Table 2.** Significant results from RM-ANOVAs in turn-level analysis.

Channel number	MNI	Neural localization	Observe > perform	Interaction	Observe > baseline	Baseline > perform	Perform > baseline
2	−52–68 41	Left angular gyrus	DX: *F* = 14.881, *p = *0.002		OX: *F* = 2.343, *p = *0.004	DX: *t *= −2.737, *p *= 0.017	
3	−67–33 32	Left anterior IPL					
4	−57–45 47	Left posterior IPL			TX: *t *= 2.772, *p *= 0.016		
5	58–47 50	Right posterior IPL		DX: *F* = 6.615, *p = *0.023	OX: *F* = 3.441, *p *= 0.004; TX: *t *= 3.25, *p *= 0.006		OX: *t *= 2.499, *p *= 0.027; TX: *t *= 2.959, *p *= 0.011
6	67–37 36	Right anterior IPL		**OX: *F* = 5.893, *p *= 0.03;** **DX: *F* = 8.608, *p = *0.012**			
7	50–69 43	Right angular gyrus	**OX: *F* = 6.109, *p *= 0.028;** **DX: *F* = 8.116, *p = *0.014**	DX: *F* = 6.732, *p = *0.022	OX: *F* = 4.653, *p *< 0.001; TX: *t *= 3.803, *p *= 0.002		OX: *t *= 2.644, *p *= 0.02; TX: *t *= 2.899, *p *= 0.012
8	60–60 28	Right TPJ	DX: *F* = 5.985, *p = *0.029		OX: *F* = 4.537, *p = *0.001; TX: *t *= 3.949, *p *= 0.012		OX: *t *= 2.897, *p *= 0.012; TX: *t *= 3.421, *p *= 0.005

OX: oxy-Hb; DX: deoxy-Hb; TX: total-Hb. Bolded cells reflect results common to both oxy- and deoxy-Hb.

### Discussion

The aim of this paper was to explore neural mechanisms involved in observing and imitating irrational actions. Participants observed and were able to imitate actions with high and low curvature (irrational and rational, respectively) while brain activity in parietal cortex was recorded with fNIRS. We found small imitation effects in behavior. Right angular gyrus and left TPJ were more active when observing actions compared to performing actions. Right aIPL was most active when participants were specifically observing irrational actions. However, the rationality effect on performance for this task was fairly weak. This effect was likely too weak to lead to observable neuroimaging differences. We discuss the implications of these data for future research into imitation.

### Behavioral data

As expected, participants increased the curvature of their movements after observing movements with higher curvatures. Typical adults rate curved actions as less rational than straight actions (Csibra & Gergely, 2009; Jastorff et al., 2011; Marsh et al., 2014c), so the curved actions in this task can be considered irrational. This is compatible with previous studies showing that typical adults imitate action trajectories that are considered to be irrational, therefore overimitating (Wild et al., 2009). However, the magnitude of the imitation effect was very small – while the demonstrator actions differed by 0.221 between the straight and curved, the participant actions differed only by 0.014. This again is similar to previous studies (Wild et al., 2009), and may arise for two reasons. First, participants are not instructed to imitate curvature, and only do so implicitly. Implicit mimicry is always likely to be more subtle than explicit copying. Second, overimitation is very much a social behavior, which occurs most commonly in social context such as being watched by an experimenter (Nielsen & Blank, 2011). To maintain tight experimental control, the social context of the current study was minimal. Participants saw the actions of another person on the screen in terms of mouse movements, but did not see a full person or even an acting hand. They did not have the opportunity to engage socially with another person during the task. It would be interesting in future studies to explore overimitation with live actors providing the demonstration and to manipulate social factors in more detail.

### Activations when observing actions

In our data, the channels over left TPJ and right angular gyrus both showed stronger activation when observing actions compared with performing actions. Previous studies suggest that TPJ is more active when participants observe irrational actions, possibly interpreting the intentions of the actor after IPL identifies actions as unusual (Marsh et al., 2014c). There is also right TPJ engagement when participants observe actions that are incongruent with their expectations (Pelphrey, Singerman, Allison, & McCarthy, 2003; Saxe, Xiao, Kovacs, Perrett, & Kanwisher, 2004). However, the effect we report was a general effect of observing actions, rather than a specific effect of rationality, and was lateralized to the left rather than the right. Thus, the straightforward conclusion is that left TPJ may have a role in understanding actions but perhaps not in their performance. This contrasts with the typical role of mirror regions (more anterior in parietal cortex) which are active for both performance and observation.

A similar pattern was seen in right angular gyrus, with stronger engagement for observation of action than for performance. Neuropsychological evidence has linked the angular gyrus with generating internal representations of motor actions before they are performed (Sirigu et al., 2003) and for being more active when observing objects (Moore & Price, 1999). Increased angular gyrus activity during the observation phase of the experiment could signal the participants forming representations of their future actions and how they interact with objects.

### Interaction of performance and rationality

Data recorded from the right aIPL channel showed an interaction between performance and rationality, with the strongest neural activation when observing irrational actions and weaker signals in all other conditions. Previous studies also implicate the IPL in detection of irrational actions (Jastorff et al., 2011; Marsh & Hamilton, 2011; Marsh et al., 2014c). Based on findings from Frey and Gerry that right parietal activation correlates with imitation performance (Frey & Gerry, 2006), we might have expected aIPL to have a role in both observation of irrational actions and also in execution of actions, but our data do not show this. Rather, aIPL seems to be specific to observing and understanding action rationality, with other regions having a role in execution. This illustrates the value of being able to record data during both observation and execution, to better probe mechanisms of imitation.

### Limitations

There are a number of limitations to our data. First, our experimental task was a simple mouse movement task with limited social context – stronger overimitation effects might be found with a more engaging task. Nevertheless, it is encouraging that despite limited social engagement we were able to find activation of right parietal cortex when observing irrational actions, replicating previous fMRI results. It is surprising, however, that we did not find any contrasts with baseline or any effects of performance. Considering our baseline task was merely passively viewing pictures, this should provide an appropriate baseline to compare to the active conditions. Therefore, parietal activity would be expected to be higher than baseline in the observation phase, especially in right aIPL where the results show greater activity in observation compared with performance. Effects of performance were similarly muted compared with baseline despite motor activity, such as finger tapping, typically activating IPL (Jäncke, Loose, Lutz, Specht, & Shah, 2000).

Behaviorally, the low fidelity of imitation in this task meant that participants were not performing actions of an equivalent rationality as the ones observed. This reduced the differences between participants’ curvatures and made the distinction between straight and curved performance in the brain less clear. Stronger behavioral effects would likely have given stronger patterns of parietal activation when performing than those seen in the results.

There are also some practical limitations to fNIRS. Due to the issues in recording data from participants, the sample size was far smaller than was ideal. This was worsened by the need to exclude participants whose channel locations were too far from the group mean. Although fNIRS is a very useful tool, it is reliant on measurements being taken from consistent brain regions across all participants. Many studies assume that consistent cap placement on all participants leads to recording from the same brain regions but we have seen that this is not the case. We were able to perform an accurate post hoc localization of each optode, excluding any participants whose channel locations deviated too far from the group mean. While this is somewhat inefficient in terms of data collection, it does ensure good consistency of the cortical localizations. Future studies might benefit from real-time monitoring of optode placement or from better normalization of data across participants, taking different placements into account.

### Broader implications and future directions

This study is important in showing proof of principle for several things. Firstly, fNIRS data can be recorded while a participant is performing an imitation task. This shows the potential for fNIRS for use in social cognition tasks and, more generally, as a more motion-resistant alternative to fMRI. This is emphasized by the replication of increased parietal activity when observing irrational actions shown in the fMRI literature (Marsh et al., 2014c). Secondly, it showed the variance in brain regions being recorded from even when cap placement is the same across participants, emphasizing the importance of cortical localization. While in this task, this was analyzed post hoc there is a clear and obvious advantage in using real-time spatial localization when placing the cap. Finally, we have presented a new method for robust multiple comparisons correction that is more appropriate than a Bonferroni correction in fNIRS. This can be used in future fNIRS studies.

Future studies can extend this work to examine different social contexts, such as the effects of power and status on overimitation or neural responses to social rewards. More complex motor tasks can be assessed in a similar way to the task in this study. These can involve many different actions in a chain, potentially requiring a participant to walk around a room to perform. It would be important for future studies to incorporate general physiological monitoring, such as heart rate and breathing, to disentangle general systemic hemodynamic changes from those of solely neuronal origin. This is especially true for studies with more active tasks. Moreover, fNIRS systems with greater scalp coverage than what was available for this study would also be advantageous as more brain regions can be explored, for example mPFC, which can provide a more complete understanding of the processes occurring.

### Summary

This study involved a unique adaptation of an overimitation task coupled with the use of a novel functional imaging technique in fNIRS. This task showed that overimitation can be produced by adult participants using tasks that have limited social engagement. The response in right aIPL to observing irrational actions supports previous literature. This increased activity cannot be attributed to the addition of movement to the task as the increase was seen in the observation phase instead of the performance phase, adding more support to the use of fNIRS. This is also evidence that fNIRS can be used as an alternative to fMRI when using a more motor-intensive task.
